# Application of immunofluorescence assay and nested polymerase chain reaction for query fever diagnosis in animal handlers of Puducherry, South India, and phylogenetic analysis based on IS1111 repetitive gene element

**DOI:** 10.14202/vetworld.2019.1769-1774

**Published:** 2019-11-13

**Authors:** Jothimani Pradeep, Selvaraj Stephen, Balakrishnan Sangeetha, Prabakar Xavier Antony, S. Amsaveni, Pratheesh Pooja

**Affiliations:** 1 Department of Microbiology, Mahatma Gandhi Medical College and Research Institute, Sri Balaji Vidyapeeth (Deemed to be University), Puducherry, India; 2 Department of Veterinary Microbiology, Rajiv Gandhi Institute of Veterinary Education and Research, Puducherry, India; 3 Central Interdisciplinary Research Facility, Mahatma Gandhi Medical College and Research Institute, Sri Balaji Vidyapeeth (Deemed to be University), Puducherry, India

**Keywords:** *Coxiella burnetii*, immunofluorescence assay immunoglobulin G, Q fever in Puducherry, query fever nested polymerase chain reaction, zoonosis

## Abstract

**Background and Aim:**

Diagnosis of query fever (QF) is mostly done on the basis of serological/molecular tests, due to the stringent requirement of biosafety level-3 containment facilities for isolating *Coxiella burnetii* in culture. QF is an important zoonosis and is considered to be an occupational hazard to livestock handlers. This report describes our study on the serological as well as molecular evidence of QF in animal handlers from Puducherry and surrounding Tamil Nadu, from where, to the best of our knowledge, no such reports are available so far.

**Materials and Methods:**

Seventy-five animal handlers were recruited, comprising veterinarians, slaughterhouse workers, butchers, and animal attendants of various government veterinary clinics from Puducherry and surrounding areas of Tamil Nadu state. QF serology was performed to identify Phase I and Phase II immunoglobulin G antibodies to *C. burnetii*. Nested polymerase chain reaction (N-PCR) was carried out to detect *C. burnetii* DNA in buffy coat samples by targeting IS1111 gene element. N-PCR-positive samples were sequenced and phylogenetic analysis was performed using MEGA software version 10.0.

**Results:**

A total of 21 animal handlers (28.1%) were positive for either serology or PCR. PCR alone was positive in 10 (13.4%), only serology was positive in 8 (10.7%), and both serology and PCR were positive in three samples (4.0%). GenBank accession numbers were obtained for 13 N-PCR-positive samples (MG548608-MG548620). Six of our study sequences showed close similarity with the reference isolates from Bengaluru, Colombia, Brazil, France, and Iran.

**Conclusion:**

A significant percentage of QF positivity in animal handlers of this part of South India, Puducherry, warrants a prospective study with follow-up of a large number of this occupational group.

## Introduction

Query fever (QF) is an important emerging zoonotic disease worldwide [1,2] but does not come under the list of notifiable diseases in India and several other countries [3]. It is caused by *Coxiella burnetii* and is an occupational disease to animal handlers such as veterinarians, butchers, and slaughterhouse workers in abattoirs/animal farms, but mostly, they are asymptomatic. The disease can be transmitted to humans by either ingestion of unpasteurized milk or inhalation of abortion products of domestic animals [2,4-11].

Only a few reports of *C. burnetii* isolation from aborted tissues and blood samples of livestock have been published in Indian literature [12-15]. Since isolation in culture is confined only to reference laboratories due to biosafety concerns, serology/polymerase chain reaction (PCR) is considered to be the preferred test [2]. Several studies from India have employed serological tests for the detection of *C. burnetii* antibodies in blood samples of domestic animals as well as humans [3,14,16-19]. In India, few researchers performed PCR for confirming this zoonosis [3,12,15,17] and the phylogenetic tree was analyzed on the basis of IS1111 gene target [13]. To the best of our knowledge, QF in this occupational category has not been reported in South India so far.

The objective of this preliminary research was to study the prevalence of QF in animal handlers of Puducherry by applying the gold standard serological test immunofluorescence assay (IFA) and the molecular diagnosis by performing nested PCR (N-PCR).

## Materials and Methods

### Ethical approval

This study was conducted in a tertiary care teaching hospital, Puducherry, with approval from the Institution Human Ethics Committee.

### Study area

This study was conducted in the department of microbiology of a tertiary care superspecialty teaching hospital and genomics and proteomics department of central research laboratory at Puducherry during January 2015-March 2018.

### Processing of blood samples

Five mL of blood was collected from each of 75 animal handlers during January 2016-December 2017. Serum samples and DNA extracts from buffy coats were preserved at −80°C. Batch testing by N-PCR and IFA was performed after an interval of 6-12 months.

### IFA

**Figure-1:**
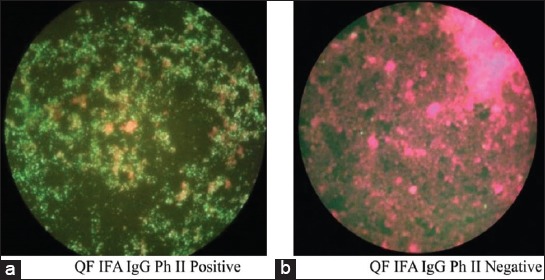
Results show the presence of query fever Phase II immunoglobulin G antibodies in a and but absent in b.

QG-120 (Phase I + II) IFA (*C. burnetii* IFA Fuller Laboratories, California, USA) was performed according to the manufacturer’s instructions. For immunoglobulin G (IgG) IFA, patients’ serum samples were diluted 1:16 using IgG Sample Diluent which contains goat serum in phosphate-buffered saline (PBS). Further dilutions were carried out in PBS for positive samples. Slides were incubated at 37°C for 30 min in a humidity chamber. Positive and negative controls were included daily during each run. Slides were removed from the incubator and gently washed with PBS, dipped in PBS for 5 min and kept in sterile distilled water to remove the residues and allowed to dry. Ten μl of conjugate, which comprises purified DyLight 488-labeled goat anti-human IgG (heavy chain) with bovine serum albumin and Evans’ blue counterstain was added to the wells and incubated in the dark for 30 min. Slides were gently rinsed with PBS for 3 times and allowed to dry. After the final wash, the slides were mounted with the mounting medium and read with 400×, at 390 nm using Primo Star iLED fluorescent microscope (Carl Zeiss MicroImaging, GmbH, Gottingen, Germany). Figure-1a shows apple-green fluorescence in a red background for positive samples and Figure-1b without any green fluorescence. According to the kit, significant titer for IgG Phase I is ≥1:16 and for IgG Phase II, it is ≥1:256. However, we took the cutoff titers for Phase-II/Phase-I IgG ≥1:128 which were considered positive for QF as per CDC criterion [1].

### DNA extraction

The genomic DNA was extracted from the buffy coats using QIAGEN Blood Mini Kit (QIAGEN, Germany). The procedure was carried out as per the manufacturer’s instructions. The purity of extracted DNA samples was checked by Eppendorf BioSpectrometer^®^ basic (Eppendorf, India). The genomic DNA was aliquoted and stored at −80°C until further use.

### N-PCR

N-PCR was carried out targeting IS1111 transposon repetitive gene element with primers developed by Lorenz *et al*. [20]. N-PCR primers were standardized using gradient PCR and the procedure was performed.

### N-PCR amplification protocol

Both N-PCR amplifications were carried out in 25 μl of reaction mixture with 12.5 μl × 2 Taq DNA Polymerase PCR kit (Ampliqon), 1 μl of forward primer and reverse primer each, 2 μl template DNA, and 8.5 μl molecular grade distilled water. The first round amplified products were used as a template for the second amplification. Briefly, the cyclic conditions for the first amplification were carried out using outer primers (Trans 1 and Trans 2) with initial denaturation at 95°C for 8 min followed by 35 cycles of denaturation 95°C for 30 s, annealing at 63°C for 40 s, and the last cycle extended for final extension at 72°C for 5 min. The second amplification was carried out using inner primers (Trans 3 and Trans 4), which was similar to the first amplification except in the annealing temperature at 61°C for 40 s (instead of 63°C). Details of the primers are as follows:

Trans 1: 5’-TATGTATCCACCGTAGCCAGTC-3’,

Trans 2: 5’-CCCAACAACACCTCCTTATTC-3’,

Trans 3: 5’-GTAACGATGCGCAGGCGAT-3’, and

Trans 4: 5’-CCACCGCTTCGCTCGCTA-3’.

The first set of amplification for outer (Trans1 and Trans 2) primers was observed at 687 bp and the second amplification for inner primers (Trans 3 and Trans 4) was observed at 243 bp.

N-PCR was carried out using Veriti Dx 96-well Thermal Cycler, Applied Biosystems (Thermo Scientific). The amplified products of amplicon size were visualized at 243 bp in 2.0% agarose gel electrophoresis with ethidium bromide solution. N-PCR-positive samples (243 bp) were purified using QIAquick PCR Purification kit (Qiagen, Germany). Unidirectional gene sequencing was done by Eurofins Genomics India Pvt. Ltd., Bengaluru, India. These sequences were aligned with MultAlin software and submitted to the GenBank, NCBI database. The phylogenetic tree was constructed using MEGA software version 10.0 (Temple University, USA) by maximum likelihood method [21,22].

### Statistical analysis

Mean ± standard deviation with 95% confidence interval for the age of animal handlers was calculated using GraphPad QuickCalcs software, USA. Sensitivity, specificity, positive predictive value (PPV), and negative predictive value (NPV) for N-PCR were calculated with MedCalc software, Belgium, keeping IFA as the reference.

## Results

**Figure-2:**
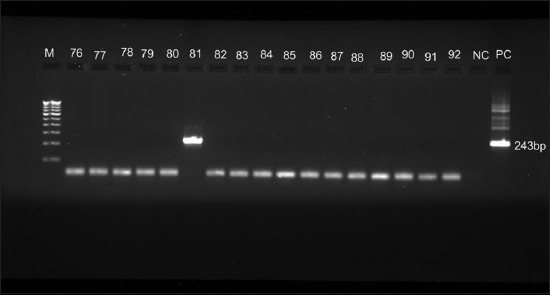
Query fever animal handlers as evidenced by nested polymerase chain reaction for IS1111gene. M – 100 bp DNA marker; Samples 76-80 and 81-92 were negative and sample 81 is positive for *Coxiella burnetii* DNA; NC: Negative control; PC: Positive control amplified at 243 bp.

**Figure-3:**
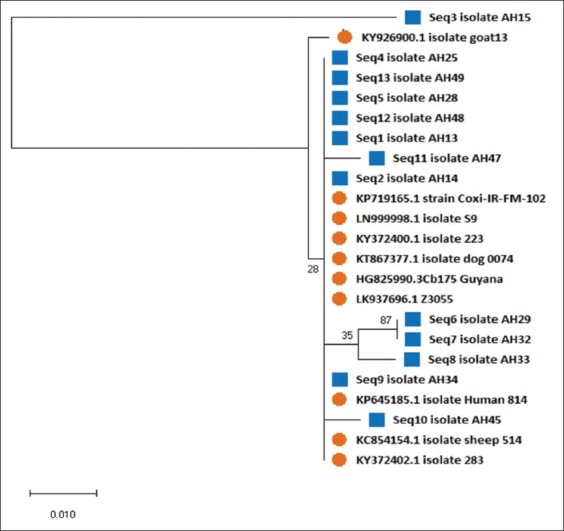
Molecular phylogenetic analysis of IS1111 gene among animal handlers. The evolutionary history was inferred using the maximum likelihood method based on the Tamura-Nei model [24]. Phylogenetic tree was constructed using MEGA software version 10.0 by maximum likelihood method. Blue color indicates our study isolates and orange indicates reference strains from the other isolates.

Animal handlers’ age ranged from 18 to 70 years and mean ± SD was 35.8 ± 13.9 years with 95% confidence interval of 32.7-39.1 years. Results of serology and N-PCR are presented in Table-1. Among 21 (28%) animal handlers with serological and/or molecular evidence of QF, PCR alone was positive only in 10 (13.4%), (Figure-2), only serology was positive in 8 (10.7%), and both PCR and serology were positive in three samples (4%). Remaining 54 (72%) were negative for both serology and PCR. Sensitivity, specificity, PPV, and NPV of N-PCR against IFA were 27.3%, 84.4%, 23.1%, and 87.1%, respectively. Gene sequencing was carried out for 13 samples in Eurofins, Bengaluru. Thirteen sequences were deposited in NCBI database and GenBank accession numbers were published (MG548608-MG548620). Phylogenetic analysis was made in IS1111 gene and shows that the six study sequences (AH25, AH28, AH49, AH48, AH13, and AH14) strongly form a cluster with the reference strains KY372400.1 (Colombia), KT867377.1 (Brazil), KP719165.1 (Iran), LN999998.1 (Bengaluru), LK937696.1 (France), and HG825990.3 (France). Within the clusters, the five isolates (AH10, AH47, AH29, AH32, and AH33) form a subclade and supported by a bootstrap value of 35% and 87%, respectively (Figure-3). Only one isolate (AH34) showed similarity with the reference isolate KP645185.1 from Brazil.

**Table-1:** QF IFA IgG Phase I/II and/or N-PCR positivity among animal handlers (n=75).

S. No.	QF IFA IgG positivity		N-PCR positivity (IS1111 gene)	Occupation

	Phase I titer	Phase II titer		
1.	1:256	1:128	Negative	Animal attendant
2.	1:128	1:512	+	Animal attendant
3.	1:128	1:512	+	Butcher
4.	1:64	1:256	Negative	Butcher
5.	1:128	1:256	Negative	Butcher
6.	1:128	1:512	Negative	Animal attendant
7.	1:128	1:256	Negative	Animal attendant
8.	1:64	1:512	Negative	Animal attendant
9.	1:256	1:128	+	Butcher
10.	1:256	1:512	Negative	Butcher
11.	1:64	1:256	Negative	Farmer
12-21.	Negative	Negative	Positive	Animal handlers
22-75.	Negative	Negative	Negative	Animal handlers

N-PCR=Nested polymerase chain reaction, QF IFA IgG=Query fever immunofluorescence assay immunoglobulin G, N-PCR=Nested polymerase chain reaction

## Discussion

Our study has identified evidence of QF in 21 of 75 animal handlers (28%) by IFA and/or PCR. According to CDC criteria, 13 N-PCR-positive samples may be considered as “laboratory-confirmed” QF cases [1]. Seven, in this group, had significant single IFA titers of Phase II IgG ≥1:128 and one with Phase I IgG titer of 1:256. These eight seropositive cases belong to the CDC category of “laboratory supportive” QF. CDC does not recommend Phase II Immunoglobulin M (IgM) testing for acute QF since IgM may persist for up to 1 year and non-specific (giving false positives) [1]. In this context, Jager *et al*. [23] recommended a cutoff IFA titer ≥ 1:32 for all the four classes of antibodies.

Altogether, 21 animal handlers (28%) had occupational exposure to and tested positive for *C. burnetii*. Blood samples were tested 6-12 months after collection. DNA deterioration in some samples might have occurred due to the delayed testing and thus, PCR positivity could perhaps be more than what we obtained. This scenario of a significant percentage of animal handlers (28%) giving evidence of prior exposure to *C. burnetii* is in contrast to our earlier experience of a low coxiellosis seroprevalence of 0.9%, 1.1%, 5.6%, and 1.85% in cattle, buffaloes, goat, and sheep, respectively [15]. It is known that seropositive animals need not necessarily shed the organism in their secretions/excretions. Conversely, seronegative animals can still harbor *C. burnetii*. Whitney *et al*. [6] reported 22.2% seroprevalence of QF among US veterinarians. Khalili *et al*. [4] reported a very high 68% seropositivity among Iranian slaughterhouse workers. Wielders *et al*. [7] from the Netherlands observed that 36% of veterinarians had Phase I IgG antibodies (≥1:1024) and considered as possible chronic QF. According to Szymanska-Czerwinska *et al*. [5] from Poland, 31.1% of the farmworkers were positive for IFA (Phase I and/or II) IgG. Park *et al*. [9] reported 9.3% of *C. burnetii* infection among workers in cattle slaughterhouse. Significance was observed among *C. burnetii* infection and carcass evisceration workers in South Korea. Seroprevalence of *C. burnetii* was estimated in 35.6% cattle farmers and farm residents of Northeastern Provinces and Inner Mongolia region of China [10]. In Afghanistan, a cross-sectional study was conducted by Akbarian *et al*. [11] and reported that 63.9% of QF seropositivity was observed among householders with their animals in the villages of Herat Province. By seroprevalence alone, 14.7% of our animal handlers were positive for QF.

Sensitivity and specificity of our N-PCR were quite low (27.3% and 84%, respectively) compared to Vaidya *et al*. [3] who reported 84.2% sensitivity and 100% specificity for their PCR against IFA. However, their subjects were women with abortion, whereas our participants were healthy animal handlers. Sensitivity of PCR/IFA depends on the time of collection of blood. PCR detects *C. burnetii* DNA in early stage after the onset of clinical symptoms, usually during the first 3 weeks before seroconversion. According to Schneeberger *et al.*, PCR becomes negative after the appearance of antibodies [24]. Phase II antibodies (IgM and/or IgG) are more predominant in early stage of the disease [1,24]. Phase II IgM antibodies may appear during 10-17 days of acute febrile illness, with simultaneous detection of Phase II IgG antibodies. IgM Phase II may be present even up to 1 year and IgG Phase II could be detected even for much longer period [1]. Hence, assessing the performance of molecular tests keeping the gold standard serological test, IFA is questionable since these are two entirely different parameters. Simultaneous detection of *C. burnetii* DNA and Phase II IgG antibodies may probably due to repeated exposure to infected livestock.

Trans-PCR targets the highly sensitive and specific gene IS1111, a transposase repetitive gene element. It is a multicopy genome, which is present in *C. burnetii*. It is highly helpful in the identification of *C. burnetii* DNA in different clinical specimens. This trans-PCR is essential to identify the positive samples, purify them, and perform gene sequencing, which ultimately will help in the construction of phylogenetic tree construction. Phylogenetic analysis of IS1111 gene of our study isolates shows maximal identity of 99% with the reference isolates, particularly from Bengaluru, Brazil, Colombia, France, and Iran.

To the best of our knowledge, animal handlers were not specifically examined for *C. burnetii* antibodies in India so far. This preliminary study was aimed at screening the animal handlers for QF with single but not paired serum samples. The mere presence of *C. burnetii* antibodies, without any clinical symptoms of QF does not require antibiotic treatment [1]. Now with current evidence of prior exposure to *C. burnetii* in 21 animal handlers, further elaborate work is warranted, by extending the study to include a larger population. Both clinical and laboratory investigations of these occupational exposures are to be carried out in long term. Animal handlers should be educated to prevent the infection by means of using protective clothing and proper disposal of animal waste is needed.

## Conclusion

In spite of occupational hazards for animal handlers to *C. burnetii*, the awareness among them is minimal. We have provided retrospective evidence of QF in population of Puducherry. Further research is therefore needed to study the extent of this zoonosis in animal handlers of Puducherry.

## Authors’ Contributions

SS, BS, JP, and PXA planned, designed, and conducted the study. BS and JP performed sample collection. JP, PP, SS, BS, and PXA carried out data analysis. SA and JP performed the phylogenetic analysis. SS and PP drafted and revised the manuscript. All authors read and approved the final manuscript.
